# Social Determinants of Health and Child Maltreatment Prevention: The Family Success Network Pilot

**DOI:** 10.3390/ijerph192215386

**Published:** 2022-11-21

**Authors:** Michelle Johnson-Motoyama, Deborah Moon, Nancy Rolock, David Crampton, C. Bailey Nichols, Hanna Haran, Yiran Zhang, Yasuyuki Motoyama, Eric Gonzalez, Nicole Sillaman

**Affiliations:** 1 College of Social Work, The Ohio State University, Columbus, OH 43210, USA; 2 School of Social Work, The University of Pittsburgh, Pittsburgh, PA 15260, USA; 3 Jack, Joseph and Morton Mandel School of Applied Social Sciences, Case Western Reserve University, Cleveland, OH 44106, USA; 4 City and Regional Planning of the Knowlton School, The Ohio State University, Columbus, OH 43210, USA; 5 The Ohio Children’s Trust Fund, Columbus, OH 43215, USA

**Keywords:** social determinants of health, interventions, programs, child maltreatment, prevention

## Abstract

Child maltreatment is a highly prevalent public health concern that contributes to morbidity and mortality in childhood and short- and long-term health consequences that persist into adulthood. Past research suggests that social determinants of health such as socioeconomic status and intergenerational trauma are highly correlated with child maltreatment. With support from the U.S. Children’s Bureau, the Ohio Children’s Trust Fund is currently piloting the Family Success Network, a primary child maltreatment prevention strategy in Northeast Ohio that seeks to address these social determinants through pillars of service that include family coaching, financial assistance, financial education, parenting education, and basic life skills training. This study highlights the initial development phase of a pilot study. Plans for in-depth process and outcome evaluations are discussed. The project seeks to improve family functioning and reduce child protective services involvement and foster care entry in an economically disadvantaged region.

## 1. Introduction

Social determinants of health (SDOH) are defined as “conditions in the place where people live, learn, work, and play that affect a wide range of health and quality-of-life risks and outcomes” [1]. Health conditions and outcomes affected by SDOH are many, which include but are not limited to diabetes [2], lower back pain [3], dental health [4], medication adherence [5], mental health outcomes [6], and more. Socioeconomic status (SES), a key domain of SDOH, contributes to health inequities through differential access to material resources, the ability to obtain healthcare services, and lifestyle behaviors [7]. Low SES has been linked to higher mortality rates [8] as well as an increased risk for myriad health issues including food insecurity, mental illness, and substance use, risk factors associated with child maltreatment [9].

Child maltreatment (i.e., child abuse and neglect) is a public health problem that contributes to morbidity and mortality in childhood and increased risk for health concerns into adulthood such as alcoholism, smoking, and drug abuse; depression and suicide; high-risk sexual behaviors; sexually transmitted diseases; and certain chronic diseases [10,11]. In the U.S., an estimated 37.4% of children experience a child protective services (CPS) investigation in response to a referral for child maltreatment by their 18th birthday [12]. During fiscal year 2020 alone, CPS agencies in the U.S. received an estimated 3.9 million referrals involving approximately 7.1 million children. Of these children, an estimated 618,000 were determined to be victims of child maltreatment, a rate of 8.4 victims per 1000 children [13]. A costly public health problem, the total lifetime economic burden resulting from just one year of fatal and non-fatal confirmed child maltreatment in the United States has been estimated at USD 124 billion [14] with lifetime costs for survivors comparable to Type 2 diabetes [15].

Social and community contexts are other important SDOH that are relevant to the quality of human interactions and relationships within the immediate family as well as in the larger community environment [16]. In particular, negative parent–child relationships, harsh discipline, and child maltreatment can cause serious harmful effects on child development as well as various health outcomes that persist throughout a lifetime [17,18]. Moreover, those harmful effects can be passed down to the next generation through intergenerational trauma, significantly affecting population health [19,20,21,22]. While not yet conclusive, a substantial body of evidence indicates that parenting and family relationships have been proposed as mechanisms through which such generational transmission can occur [23,24,25,26]. Thus, promoting positive parent–child interaction is one of the major objectives that Healthy People 2030 initiatives are focusing on to improve population health by making positive changes in the social and community context [27].

In the effort to tackle various SDOHs to improve population health, it is important to consider intensified adversities for families affected by multiple SDOHs. Parents who were maltreated as children and live with unaddressed trauma may struggle with emotional dysregulation, maladaptive coping skills, and difficulties with interpersonal relationships. The added stress resulting from poverty may further compromise such parents’ ability to provide healthy and nurturing relationships for their children, contributing to the cycle of maltreatment [28].

In this article, we describe the Family Success Network (FSN), an intervention that was conceptualized, developed, and implemented to address heightened risks for child protective services involvement stemming from neglect, which is often associated with intergenerational trauma, poor socioeconomic conditions, and a lack of resources for prevention. Researchers, policy makers and service providers are looking for tested models that provide an alternative to child welfare for families who may benefit from family support. In this paper, we provide an overview of the FSN program and describe the steps a cross-sector community collaborative engaged in to conceptualize the service. We then provide an overview of a target area needs assessment the authors engaged in to support FSN outreach and engagement in the tri-county area, discuss the evaluation, and offer directions for future implementation and evaluation.

## 2. The Family Success Network (FSN)

The FSN is a multicomponent community-based child maltreatment prevention pilot that is currently being implemented in three under-resourced counties in Northeastern Ohio with support from the Administration for Children and Families, Children’s Bureau, *Community Collaborations to Strengthen and Preserve Families* grant. A key component of the FSN is that it provides families and community members with a place where they can seek support, without the stigma and fear that is typically associated with CPS involvement.

The three-county area included approximately 529,000 people in 2019. Geographically, the area includes pockets of Appalachia as well as the city of Youngstown (population 60,000). Compared to other Ohio counties, the three counties have relatively high poverty rates: approximately 18% of people in Mahoning and Trumbull Counties and 14.5% of people in Columbiana County live under 100% of the federal poverty line. All three counties experienced population growth until the 1980s, which was subsequently followed by a precipitous population decline from 2000 to 2019. Today, the three counties have the lowest population growth rates in the state [29].

In response to the diverse needs and challenges facing families in the area, the FSN was conceptualized by the Ohio Children’s Trust Fund in partnership with a number of state and community partners within each county (i.e., community collaboratives). The community collaboratives conducted a comprehensive regional needs assessment in 2016 to identify the scope of societal problems related to child maltreatment, as well as address service gaps within the child welfare continuum of care. The needs assessment identified protective and risk factors across various ecological levels including the child, family, community, and society. Data from the state’s child protective services agency revealed that while the total number of child maltreatment allegations in the Northeast Ohio region had increased at a slightly higher level than the rest of the state over time, many of the families who were subjects of these maltreatment reports could benefit from community-based prevention services to increase protective factors and prevent involvement with the formal child protection system. Despite the numerous risk factors presented, a key strength noted in the needs assessment was the well-established collaborative relationships that existed within each county, which uniquely positioned the FSN to implement a program model that would address many of the challenges and needs identified within the Northeast region of the state.

**Figure 1:**
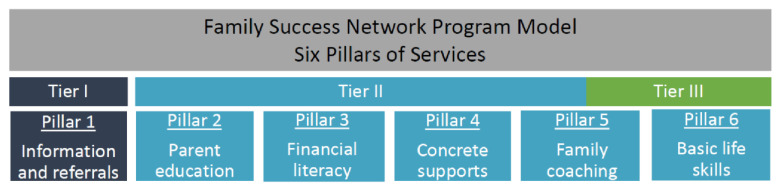
The Family Success Network Program Model.

Members of the community collaboratives defined the root causes of child maltreatment in the three counties as intergenerational maltreatment, poor parenting practices, limited household resources for parenting, and a gap in a single access point for child maltreatment prevention services. In response, they designed a three-tiered intervention that occurs outside the CPS system in an effort to reduce the stigma associated with CPS involvement. Services and supports are provided by community-based agencies that coordinate service provision within the community to provide the following types of services and supports: (1) family coaching, (2) parenting skill-building; (3) basic life skills; (4) financial literacy; (5) concrete supports; and (6) information and referrals to strengthen families and prevent child maltreatment (Figure 1).

The main components of the FSN focus on parent–child relationships and financial needs to address intergenerational trauma and buffer immediate financial hardships. Tier I focuses on the provision of information and referral services to connect participants to community resources. Tier II encompasses Tier I services in addition to parent education, financial literacy, concrete supports (e.g., material goods, transportation tokens, financial assistance, etc.) and family coaching. Tier III is the most comprehensive and provides family coaching, home visits and basic life skill support in addition to Tier I and II services. All services are voluntary, and families may fluidly move between Tier II and Tier III depending on their preferences for the level of engagement with the program and their family needs. Parenting skill-building is addressed through the Triple P parenting program. Triple P provides parents with education and support for children’s behavioral and social development by equipping parents to address the behavioral problems of their children while concurrently strengthening parent–child relationships. Additionally, intergenerational trauma and relational health are addressed through family coaching and assessments of past family traumas using the Adverse Child Experiences (ACE) Study [30], the Brief Child Abuse Potential Inventory (BCAP) [31], and the Protective Factors Survey 2nd Edition (PFS-2) [32].

The family coaching component is viewed as the intervention’s primary mechanism of change and consists of solution-focused coaching sessions designed to engage families in moving towards behavioral change and positive relational health using motivational interviewing techniques. The family coaches often share life experience with the families receiving services, which facilitates trust, rapport building, and engagement with services.

The Transition to Independence Process (TIP) model [33] is an evidence-supported practice that was adapted to promote basic life skills among FSN participants. TIP engages participants to plan for their future by providing them with trauma-informed services and supports. The TIP model uses five Transition Domains to track improvements, engagement and progress in education where appropriate; the acquisition of relevant life skills for daily functioning; and changes in interpersonal skills, mental health and/or substance use problems, and the stability of living situations..

Economic support is provided by the FSN through financial literacy and concrete support and financial assistance up to USD 500. Financial education is offered through Money 101, a financial literacy program [34]. Money 101 helps participants develop foundational financial literacy skills including setting up a budget, tracking spending, financial goal setting, and identifying action steps to improve credit scores. Concrete supports include diapers, wipes, gas cards, and bus passes to reduce financial stressors that may be affecting parenting and relational health. Families who participate in Money 101 are also eligible to receive financial assistance to pay past due utility bills, rent payments, or address other emergency financial needs. While the amount of financial assistance may seem limited at first glance, past research suggests that even small amounts of money can have preventative effects on child maltreatment [35].

## 3. Target Area Needs Assessment

To support the FSN in their outreach and engagement efforts, the evaluation team developed a Target Area Needs Assessment (TANA), which included a review of quantitative child maltreatment data and an environmental scan of services and community partners. Quantitative data for the TANA were drawn from publicly available sources, including the U.S. Census Bureau American Community Survey, the Annie E. Casey Foundation and others [36] to further illustrate the socioeconomic context of the three counties as well as demographics including child race and ethnicity. According to 2020 estimates, the majority of children in all three counties were White with larger populations of Black children in Mahoning (25%) and Trumbull (14%) counties (see Table 1).

In addition to county demographics, the project was interested in understanding the needs within each county. To address this concern, the evaluation team produced zip-code level maps of child protective services referrals. With these color-coded maps and background information, the evaluation team engaged in discussions with the community collaboratives in each county. These discussions revealed the nuances of socioeconomic conditions in different areas of each county, identified particularly underserved populations, and stimulated useful conversations about community partnerships and culturally responsive outreach in high child maltreatment referral areas. For example, one discussion revealed the presence of a sizeable immigrant Guatemalan population in Columbiana County, a community of predominately white, Appalachian residents. The group noted that the schools and at least one church had strong connections to this Guatemalan community and the FSN could partner with them to reach these families. Other conversations revealed important cultural considerations in outreach to Appalachians in Columbiana County while African American churches with youth programs were highlighted as an important resource for engagement in Trumbull County. To support efforts to reach the diverse populations residing in the tri-county area, the FSN hosts monthly discussions organized around research reviews prepared by the evaluation team on relevant engagement topics.

## 4. Looking Forward: Evaluation

The evaluation team is currently engaged in evaluation activities to assess FSN program effectiveness and to facilitate replication of the program in other geographic areas. The outcome evaluation includes participant-level, community-level and system-level components. At the participant level, we are examining the efficacy of the FSN using a randomized wait list control group design. All evaluation participants complete pre-test measures including the ACEs, BCAP, PFS-2, and the Relationship Quality Index (RQI) [37] and are then randomized into the FSN program or a three month wait list control group with concrete services and financial education. The randomization design is unbalanced to maximize service delivery. Of every four participants that agree to take part in the study, one is asked to wait three months for the full suite of FSN services. Treatment group participants are asked to complete the Working Alliance Inventory (WAI) [38] after two weeks of services to gauge the quality of the relationship between the participant and the family coach. Post-test data are collected for treatment and control groups three months after baseline data collection to assess changes in child maltreatment risk, protective factors (family functioning/resilience; nurturing and attachment to children; social supports; and concrete supports), relationship quality with an intimate partner, and relationship quality with the family coach. In addition, participant data are linked to administrative data to determine whether participants are served by child protective services during or after receiving FSN services.

At the community level, we plan to compare changes over time in children’s services referrals accepted for investigation and foster care entries in a set of comparison counties in Ohio that will be identified through a cluster analysis using historical child welfare data. Additionally, we will examine trend data over time in CPS investigations and foster care entries in target area zip codes before and after FSN implementation. At the system level, we will use a pre-post design to evaluate system level outcomes tracked using the Collaboration Assessment Tool [39], which will be administered annually in combination with questions that address the project’s system-level outcomes.

Along with the outcome evaluation, we are conducting a process evaluation to develop deeper insights into the aspect of FSN implementation and how the implementation factors might have contributed to the program outcomes. The process evaluation examines three main areas of implementation including reach, fidelity, and implementation drivers.

To explore the program’s reach, we are examining the number and representativeness of families who participated in each tier and the six pillars of services provided through FSN. Additionally, we will examine the characteristics of family participants through descriptive analyses, including demographic factors, types of family needs and concerns as well as baseline protective factors and maltreatment risk.

Fidelity assessment focuses on examining how individual program components and the entire FSN program were implemented as originally intended. Fidelity assessment planning was carried out collaboratively, extensively involving organizational leadership and service providers. The process entailed seven iterative steps described in detail elsewhere [40], which included (1) educating leadership and service providers while developing shared goals; (2) developing the theory of change and logic model; (3) identifying essential program components; (4) determining fidelity domains and indicators; (5) identifying methods of data collection focusing on capacity building; (6) creating a fidelity scoring system; and (7) eliciting feedback and revising as needed.

Lastly, interviews and focus groups will be conducted to explore the perspectives of organizational leadership, service providers, and the implementation advisory groups in each county concerning the facilitators and barriers to FSN implementation as well as recommended solutions. The data will inform the effort to further refine the program and replicate FSN in other regions.

## 5. Conclusions

The FSN program is a primary prevention strategy that is being piloted in three counties in Ohio. It seeks to prevent child maltreatment and CPS involvement through cross-systems collaboration and multi-tiered services that address immediate economic needs and intervene in intergenerational maltreatment by improving parent–child interactions. As an organization, the FSN implementation and evaluation teams collaborate as a learning organization to promote equity in outreach, engagement, and service provision with the intention of reducing social disparities in CPS involvement. However, the program is not without its limitations. The FSN was designed to serve families who volunteer to participate. As a voluntary program, not all families who have needs for FSN services may know about them or choose to participate. The OCTF is currently restricted from serving families with a substantiated CPS report or an active CPS case per Ohio Revised Code. Therefore, families cannot be mandated to the program. However, to enhance outreach and better serve families who may have been the subjects of CPS reports, the FSN has partnered with CPS agencies in the three counties to share information about the program. The CPS agency informs the individual who made the report about the FSN program for referral purposes. The FSN offers an array of family support services; however, the program is unable to provide additional services that might prevent child maltreatment such as case management, child care, or services to address spousal violence. While we are unable to provide evidence of effectiveness of the FSN strategy at this time, findings from in-depth process and outcome evaluations will determine the FSN’s success in improving family functioning and resilience, interrupting intergenerational trauma, and reducing CPS involvement and foster care entry. If the program demonstrates evidence of effectiveness, OCTF has discussed plans for further expansion throughout Ohio. Evaluation of the FSN in new counties will require special attention to the unique community and organizational contexts for implementation; local economic and social conditions; local referral networks and available services; and the presenting concerns of families served.

## Figures and Tables

**Table 1:** Child race and ethnicity (2020 estimates) [36].

	White	Black	Asian	Hispanic (of Any Race)
Columbiana	95%	4%	1%	3%
Mahoning	73%	25%	1%	10%
Trumbull	85%	14%	1%	4%
State of Ohio	78%	19%	3%	7%

Note that the American Indian population in Ohio, and in each of the counties was 0%.

## Data Availability

Not applicable.
